# Understanding the Mechanisms that Operate within CHIME: A Realist Review Protocol

**DOI:** 10.12688/hrbopenres.14171.2

**Published:** 2025-11-17

**Authors:** Michael John Norton, John Paul Byrne, Tina Bedenik, Michael Ryan, Catherine Brogan, David Dwyer, Killian Walsh, Éidín Ní Shé

**Affiliations:** 1 PhD Scholar, Graduate School of Healthcare Management, Royal College of Surgeons in Ireland, Dublin, Leinster, D02 YN77, Ireland; 2 Senior Lecturer, Graduate School of Healthcare Management, Royal College of Surgeons in Ireland, Dublin, Leinster, D02 YN77, Ireland; 3 Senior Post-Doctoral Researcher, Data Science Centre, School of Population Health, Royal College of Surgeons in Ireland, Dublin, Leinster, D02 YN77, Ireland; 4 Head of Mental Health Engagement and Recovery, Office of Mental Health Engagement and Recovery, St. Loman’s Hospital, Palmerstown, Dublin, D20 HK69, Ireland; 5 303 Duncreeven, Courtown Park, Catherine Brogan Consultancy, Kilcock, Kildare, W23 Y660, Ireland; 6 Involvement Centre Co-Ordinator, Recovery College South East, Greenshill, Kilkenny, R95 YYC0, Ireland; 7 Information Specialist, Library, Royal College of Surgeons in Ireland, 26 York Street, Dublin, D02 P796, Ireland

**Keywords:** CHIME, Mental Health, Personal Recovery, Realist Review, Theory Generation

## Abstract

**Background:**

Recovery originated from the civil rights movement of the 1960s/70s. However, no universally accepted definition of recovery had been constructed until 1993 when William A. Anthony suggested that recovery involved living one’s best life even with mental health difficulties. In 2011, Leamy *et al*. created CHIME [ **C**onnectiveness, **H**ope, **I**dentity, **M**eaning and purpose and **E**mpowerment]. A concept that represents the key characteristics of recovery. It derived from a literature review into recovery from psychosis. Since 2011, the literature has examined these concepts individually and collectively to understand what they are in reality. However, few studies have investigated the internal mechanisms that causes a person to move from unwellness to recovery via CHIME. As such this proposed realist review will explore how and why the mechanisms within CHIME operate in individuals recovering from mental health challenges.

**Methods:**

This review forms work package one of a PhD study into CHIME and mental health recovery in Ireland. It complies with relevant guidelines relating to realist reviews including Pawson *et al*’s. updated methodology, which consists of six phases: 1) setting up the review advisory panel and constructing initial programme theories; 2) searching for evidence; 3) selecting and appraising evidence; 4) extracting data; 5) analysing and synthesising data; and 6) ethics and dissemination.

**Discussion & Implications for Practice:**

This proposed review will address a gap in the literature on the mechanism involved in recovery from mental health challenges. Unlike other review types, a realist review is theory orientated, allowing one to answer this review question by exploring how, why, and through what circumstances individuals reach recovery through CHIME. This review will inform future work packages of this PhD study. The proposed review will be written up and submitted to a peer-reviewed journal. Dissemination outside academia will be considered.

**Registration ID:**

CRD420251038961

## Introduction

Within the past few decades, mental health services have radically transformed on a structural, cultural, and philosophical level ^1^. Today, mental health services operate on the bases of personal recovery. A type of recovery best described through William A. Anthony’s seminal 1993 work ^2^, where he states that recovery is:

*“...a deeply personal unique process of changing one’s attitudes, values, feelings, goals, skills and/roles. It is a way of living a satisfying hopeful and contributing life even with the limitations caused by illness... includes the development of new meaning and purpose in life as one grows beyond the catastrophic effects of mental illness.”* [ 2, p.21].

As such, recovery is a strengths-based and person-centred concept, as it allows one to be viewed beyond their diagnostic label to that of a unique, whole person with wants and desires like others in society ^3, 4^. Not only has personal recovery a therapeutic value, but it is also a catalyst of individual practice and systemic cultural change. Of note, in Irish services, the current mental health policy, ‘ *Sharing the Vison*,’ is underpinned by both trauma-informed practice and personal recovery ^5^. In May 2024, the Office of Mental Health Engagement and Recovery [MHER] – an office within the Health Service Executive’s national structures responsible for the implementation of recovery in mental health services – published ‘ *A National Framework for Recovery in Mental Health*’ ^6^. This framework identifies four key ingredients for creating a recovery-oriented service including:

1. The centrality of lived experience,
2. The co-production or recovery-promoting services between all stakeholders,
3. An organisational commitment to the continuous development of recovery in Irish mental health services and
4. Supporting recovery-orientated learning and practice across all stakeholder groups ^6^.

**Figure 1.**
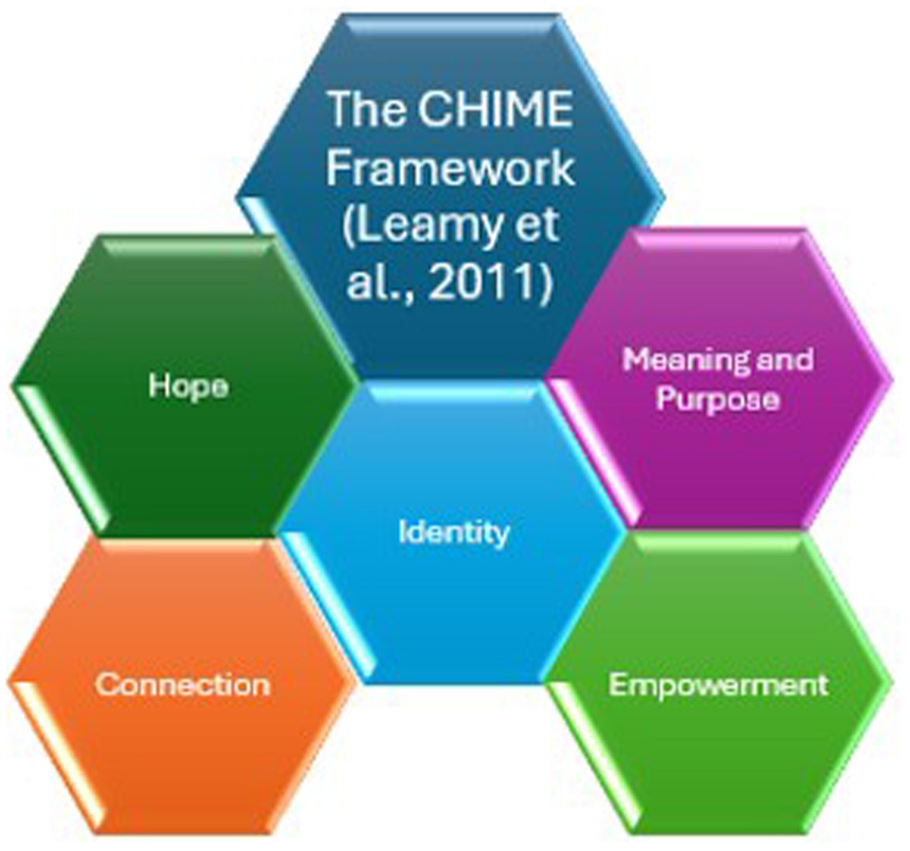
Figure depicts the five elements needed for recovery to occur. CHIME consists of the concepts **c**onnection, **h**ope, **i**dentity, **m**eaning and purpose and **e**mpowerment.

Each of these principles are necessary to create recovery-oriented services. However, to achieve recovery orientation, staff and services must first understand the concept of recovery beyond the strict limitations of a positivist biomedical lens to something that is more interpretative and dependant on the unique experiences of the individual. To do this, MHER created a suite of training on recovery known as the ‘ *Recovery Principles and Practice Workshop.’* A central tenet in this programme, which is used to support the embedding of recovery orientation, is the CHIME framework ^6, 7^ [ Figure 1].

CHIME consists of the concepts **c**onnection, **h**ope, **i**dentity, **m**eaning and purpose and **e**mpowerment. The CHIME framework was developed as a result of a systematic review and narrative synthesis of recovery in mental health for people with psychosis ^8^. Since its construction in 2011, CHIME has been utilised for research, educational, and managerial purposes. A study by Penas *et al*. ^9^ utilised the dimensions of CHIME to create a validated instrument to measure the existence of CHIME within a service. This is the closest measure available to measuring the recovery orientation of a service. In addition, CHIME is used as part of educational packages within recovery college environments to support the recovery literacy of not just staff but also service users, family members/carers/supporters, and the public at large ^10^. For example, see Recovery College West ^11^. In addition, a recent UK-based systematic review noted through a process of citation content analysis where and how CHIME is currently utilised ^12^. In addition, presently the CHIME framework has been extensively researched, particularly in anglosaxon countries where it is most widely utilised ^13, 14^. As noted above, since its construction in 2011, the concepts that make up CHIME have been explored collectively and individually ^6, 7, 9^. Additionally, CHIME has also been used to create measures of personal recovery in mental health ^9^, but also in the analysis of various interventions utilised by mental health services ^15^. In recent years, CHIME has taken on additional letters as it has advanced, for instance CHIME-D has been used with D used to represent the word disability ^16^. However, till now, no study has been formed that explicitly sets out to explore the underpinning mechanisms that formulate the concepts that are marked within the CHIME framework. The development of such a study is timely as services are now requesting guidance on how to enhance personal recovery in mental health on a micro [personal], meso [community] and macro [organisationa/societal] level ^6^. As such, such a study would be of great service value to clinicians and other healthcare professionals in enhancing recovery in mental health through CHIME.

As such, this review explores how a person moves from a place of unwellness to a place where they gain a sense of connection, hope, identity, meaning and purpose, and empowerment. In answering this question, a realist review was deemed most appropriate as it targets antecedents through questions such as who it works for, in what circumstances, why, and how ^17, 18^. Additionally, realist reviews are similar in nature and rigor to systematic reviews, but are utilised to understand the causal forces that allow a behavioural health construct to operate in one context but not in others ^19^. In recent years, realist reviews have become popular as they allow researchers to immerse themselves in these meta-theoretical spaces to explain how interventions like CHIME work ^20^. It is through exploring these meta-theoretical spaces that actions can be created, identified, and taken in order to inform policy and practice ^21^. Unlike the original systematic review and narrative synthesis that created CHIME in the first place, realist reviews permit data from a wide range of sources with various degrees of quality. Including these various data sources in the production of refined initial programme theories will support the work of recovery specialists on the ground as well as the overarching PhD study itself, as it will provide a new theoretical understanding of the mechanisms that bring people from unwellness to wellness via CHIME. The refined initial programme theories created as a result of this proposed realist review will then inform work package two of this PhD, when service users and expert panel members will be interviewed to either confirm or deny the mechanisms uncovered over the process of this proposed realist review. As such, this present paper aims to document the methodology behind a proposed realist review which *explores how and why the mechanisms within the CHIME [ **C**onnectiveness, **H**ope, **I**dentity, **M**eaning and purpose and **E**mpowerment] framework work in individuals who are in recovery from mental health challenges?*

## Methods

This protocol and the proposed realist review will comply with the **R**ealist **A**nd **M**eta-narrative **E**vidence **S**ynthesis: **E**volving **S**tandards **-I** [RAMESES-I] guidelines ^22– 24^. According to Duddy and Wong ^25^, these standards will be used to guide the reporting of realist reviews. Further details on this are discussed below. This protocol was registered with PROSPERO [CRD420251038961] on April 29 ^th^, 2025, and can be freely accessed.

### Realist review phases

Systematic and scoping reviews have a step-by-step systemic approach to the review of the literature ^26– 28^. This is important for both review types so that they can enhance the reproducibility and transparency of how papers were searched from within the wider literature base ^29, 30^. Realist reviews differ from systematic and scoping reviews as they do not conform to a prescribed method to review the literature ^18^. Instead, because of the realist’s focus on theory creation and explanation, the review process will be supported by a set of guiding principles rather than a prescribed structure ^21, 31^. As such, for the purposes of this realist review protocol, an essential step in creating the protocol was identifying what methodology to follow. This protocol followed the format proposed by Masterson *et al*. ^32^ and McCormack *et al*. ^33^. The rationale for this is twofold: 1) both studies adhered more strictly to the RAMESES-I guidance and 2) both studies were situated within either the co-design space ^32^ or within an aspect of mental health service provision ^33^. McCormack and colleagues ^33^ in particular note that a realist review comprises of five generalised steps that were originally devised by Pawson and colleagues ^34^ in their seminal work. These steps are as follows:

1. Set up of the review advisory panel and construct initial programme theories,
2. Searching for evidence,
3. Select and appraise evidence,
4. Extract the data,
5. Analyse and synthesis the data.

**Figure 2.**
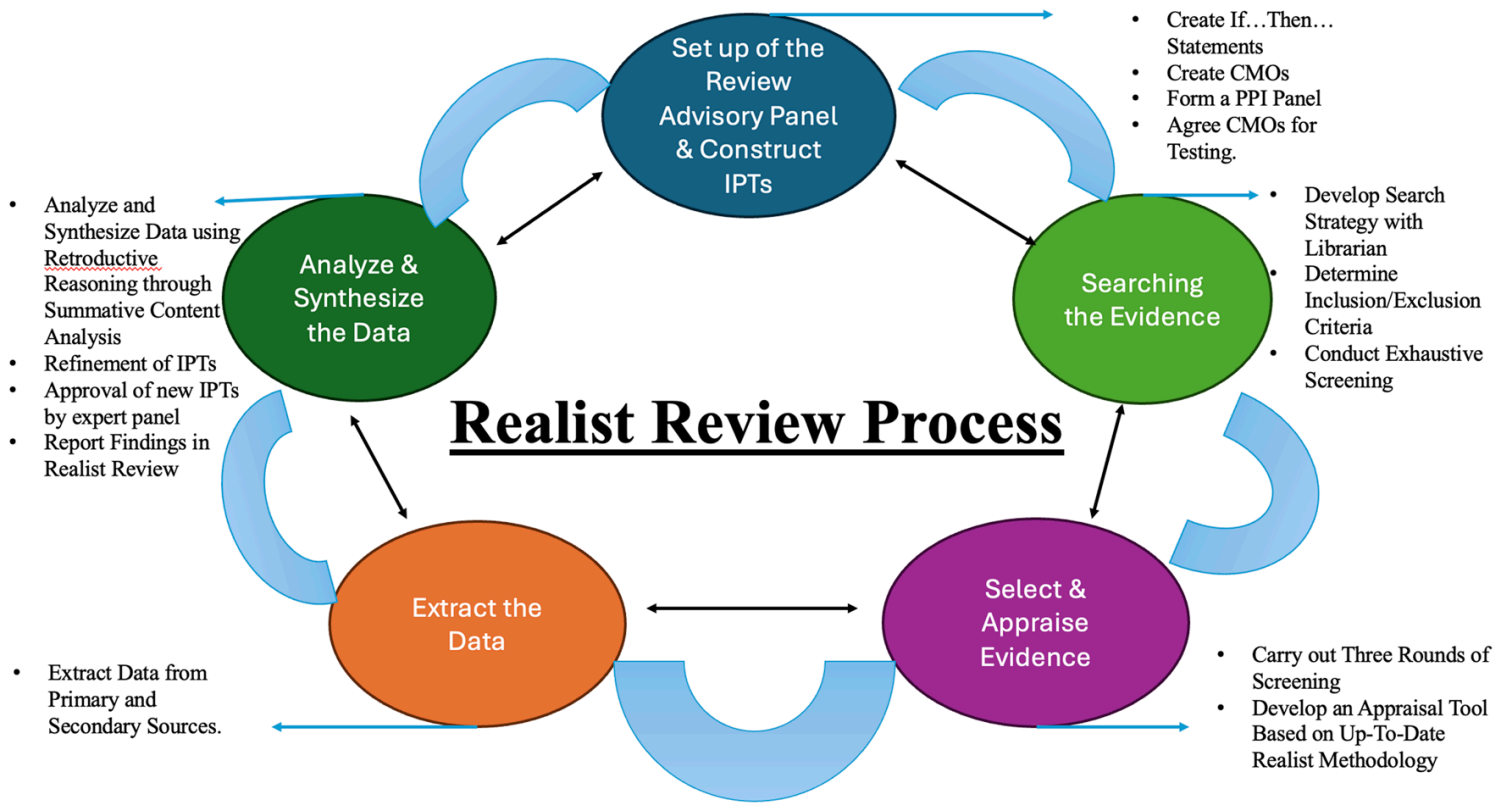
Figure depicts the five phases of Pawson and colleagues process of conducting realist reviews as adapted from the work of McCormack and colleagues.

These steps are similar to those utilised in generic systematic reviews, but they are notably different in terms of theoretical depth required to complete each step ^35^. Figure 2 illustrates the five major steps of the realist review process adapted from McCormack *et al*. ^33^. Additionally, a sixth step will be added to examine the ethics and dissemination of results, given that the present paper represents a protocol for a proposed realist review. Each step is described in detail below.

***Step one: setting up the review advisory panel and constructing the initial programme theories.*** Realist reviews are, by their very nature, are cyclical and iterative in their approach ^36^. This means that the refinement of the initial programme theories constructed here occurs throughout the review itself ^37^. In realist reviews, the first step is to identify a research focus and construct and reach an agreement regarding initial programme theories. Initial programme theories are both a theory of change and a theory of implementation and as such are iterative and subject to change. As noted previously, for this realist review, our focus will be *to explore how and why the mechanisms within the CHIME [ **C**onnectedness, **H**ope, **I**dentity, **M**eaning and purpose and **E**mpowerment] framework work in individuals who are in recovery from mental health challenges.* Given that realist reviews are explicitly theory orientated along with an emphasis on “how” and “why” within the focus statement above, this review approach is suitable for exploring the mechanisms that bring a person from a place of unwellness to the characteristics of CHIME as noted by Leamy *et al*., ^8, 25, 38^.

 The next subphase in this first step is to break down this focus into a series of “If...then...” statements ^39^. If... then statements are simply explanatory accounts or statements that allow one to begin the process of programme theorising ^40^. As noted in Table One, each element of CHIME received its own if... then... statement. This is because each element of CHIME works independently and together to support an individual’s personal recovery journey.

**Table 1.** Table consists of the five if… then… statements – the starting point in creating initial programme theories.

	Table One: If…Then… Statements
1	If a person with lived experience of mental health challenges feels connected/disconnected with themselves, others around them and with their community, then this would have a long-lasting positive/negative impact on one’s mental health recovery journey.
2	If a person with lived experience of mental illness is experiencing hopefulness/hopelessness, then this would have an impact on the person’s outlook on life, thereby impacting their ongoing, mental health recovery journey.
3	If a person with mental health challenges experiences a sense of identity/a lack of identity, beyond that of the passive service user/patient, then this would have a profound impact on the individual’s mental health condition and their sense of self-worth, which thereby impacts one’s mental health recovery journey.
4	If a person who uses community mental health services develops/loses a sense of meaning and purpose in their lives, then this can positively/negatively impact a person’s wellbeing which can influence the person’s overall mental health recovery journey.
5	If a person with lived experience of mental health challenges experiences a sense of empowerment/disempowerment, then they can feel more/less in control of their mental wellbeing, leading to healthcare choices that ultimately a positive/ negative impact on one’s treatment plan and their overall mental health recovery journey.

Once the if... then... statements are created, they are presented to the team members [ÉNS, JPB, and TB] for review and refinement. Once these if... then... statements are finalised, the next sub-step is to create the initial programme theories ^41^. Initial programme theories are useful as they help reviewers understand how, for whom, why, and under what circumstances complex interventions such as CHIME work to achieve personal recovery in individuals with mental health challenges ^42^. Essentially, an initial programme theory comes from the if... then... statements and consists of three essential parts: context, mechanism, and outcome (CMO) ^43^. In a realist review CMOs, the context refers to the outside parameters of the formal programme architecture. In other words, it relates to the environment in which the mechanism occurs ^44^. This can be in the backdrop of a historical event, or perhaps social and cultural norms ^45^. However, it is a contextual interaction with the mechanism that produces the outcome. The mechanism is the underpinning generative force that, when interacting with the context, produces the outcome. It is subdivided into resources and how people respond to them. It is often hidden and extremely sensitive to variations in context ^46^. Finally, the outcome is the result of the mechanism’s interaction with the context. These outcomes are often behavioural and can be either intentional or unintentional ^45^. In short, these components come together to be known as a CMO configuration or an initial programme theory. The premise behind a CMO configuration is that the context and mechanism lead to an outcome. This process involved multiple readings of the if...then... statements in order to clearly assign elements to the context, mechanism, and outcome. Once MJN was satisfied that these elements were aligned correctly, the CMO configurations were circulated to the supervisory team for initial analysis and refinement, resulting in an initial set of plausible programme theories, as presented in Table Two.

**Table 2.** Table consists of the original if… then… statements along with a first draft of the initial programme theories devised from these if…then… statements before review by the expert panel.

1. If a person with lived experience of mental health challenges feels connected/disconnected with themselves, others around them and with their community, whatever that community is to them, then this would have a long-lasting positive/negative impact on one’s mental health recovery journey. **Context:** A person with mental health difficulties in receipt of mental health services to support their mental health and wellbeing through regaining CHIME. **Mechanism:** Enters and/or resides in an environment that allows/inhibits a sense of connection. **Outcome:** The person feeling either a sense of connection or disconnection from themselves, others around them and with their environment leading to long lasting positive and negative effects on a person’s mental health recovery journey. 2. If a person with lived experience of mental illness is experiencing hopefulness/hopelessness, then this would have an impact on the person’s outlook on life, thereby impacting their ongoing, personal, mental health recovery journey. **Context:** A person with lived experience of having a mental illness who utilises the mental health services to support their mental wellbeing through regaining elements of wellbeing discussed within the CHIME framework. **Mechanism:** The person is, due to their ongoing mental health challenges, in a mindset that allows or inhibits a sense of hope to be created and maintained in their lives. **Outcome:** Leading the person to either feel or not feel a sense of hope, as indicating an impact on their ongoing, personal, mental health recovery journey. 3. If a person with mental health challenges experiences a sense of identity/a lack of identity, beyond that of the passive identity of a service user/patient, then this would have a profound impact on the individual’s mental health condition and their sense of self-worth, which thereby impacts one’s mental health recovery journey. **Context:** A person with lived experiences of mental health challenges who utilises mental health services for their ongoing mental health and wellbeing through reclaiming the elements of CHIME in their lives. **Mechanism:** The person has lost/gained a sense of identity to the point that the person stays within or moves beyond the passive service user/patient identity. **Outcome:** Leading to notable changes in how a person views their recovery and their measurement of their own perceived self-worth. 4. If a person who uses community mental health services develops/loses a sense of meaning and purpose in their lives, then this can positively/negatively impact a person’s wellbeing which can influence the person’s overall mental health recovery journey. **Context:** A person who avails of a community mental health service in order to reclaim aspect of CHIME needed for their ongoing mental health and wellbeing. **Mechanism:** The person, because of their engagement with community mental health services either develops or loses their perceived sense of meaning and purpose in their lives. **Outcome:** Leading to a positive/negative impact on a person’s own self-defined, personal recovery journey. 5. If a person with lived experience of mental health challenges experiences a sense of empowerment/disempowerment, then they can feel more/less in control of their mental wellbeing, leading to healthcare choices that ultimately have either a positive/negative impact on one’s treatment plan and their overall mental health recovery journey. **Context:** A person with lived experience of mental health challenges who engages with the mental health services in order to reclaim aspects of CHIME that are important for their ongoing mental health and wellbeing. **Mechanism:** The person is in a space that creates an environment where such individuals either become empowered or disempowered because of the process of being labelled as a service user. **Outcome:** Leading to feeling more/less in control of their mental wellbeing, which can lead to healthcare decisions that either benefits or challenges the person’s mental health during their own life-long, personalised recovery journey.

Once the initial CMO configurations are constructed, the next subphase is to present these CMO configurations to a group of experts for review. This is necessary in order to ensure that the theories we test the literature with are relevant to the situational context of modern mental health services, which can be determined by consulting experts in this area. From MJNs’ previous work experience, the expert panel was brought together and consisted of six individuals: three academics: Dr. Éidín Ní Shé, Dr. John Paul Byrne, Dr. Tina Bedenik, the Head of MHER, Michael Ryan, the Chair of the National Implementation and Monitoring Committee [NIMC], and former Advancing Recovery in Ireland [ARI] co-lead: Catherine Brogan and Involvement Centre Co-Ordinator and former Peer Educator: David Dwyer. In this way, the expert panel represents a diverse group of academics and local Irish experts who have a key role in implementing CHIME into practice within an Irish context. Éidín Ní Shé is a health systems academic who has a particular proficiency in realist review methodology, having published a number of realist reviews in areas of health systems management. John Paul Byrne is an academic sociologist whose interest spans the social determinents shaping work in health and psychosocial work-life conditions. Tina Bedenik is a senior postdoctural fellow with a particular interent in the ethical applications of AI in clinical decision making. Michael Ryan is a service user by background and has utilised his lived experience to support the creation and implementation of the recovery movement in Ireland. Catherine Brogan is a service provider, originally a psychiatric nurse by trade. She has utilised her practice wisdom to support, along with Michael Ryan, the creation of ARI and has for several years been a leader in creating environments that are recovery orientated within mental health. Finally, David Dwyer is also a service user by background and has utilised his lived experience to support recovery through recovery education and through the management of recovery on the ground through peer-led involvement centres. These individuals were sent an invitation letter [Appendix A] containing the above CMO configurations, along with the meeting date and associated details. Due to the proximity of all parties to one another in the country, the expert panel convened online via MS Teams and lasted approximately an hour and a half. At this meeting, the entire PhD project was presented to the expert panel, with specific attention given to work package one: the realist review. As part of this meeting, each CMO configuration was presented individually, with adequate time given for discussion of all aspects of each CMO configuration under examination. As a result, CMO configurations were added and others were refined with the support of the expert panel. After the meeting, the original CMOs were amended, and a sixth CMO was added based on the discussion. Additionally, these were added to the minutes, which were prepared and circulated among the expert panel members [Appendix B], who were then given the appropriate amount of time to respond and make further amendments to the edited CMO configurations. The finalised and amended CMO configurations are presented in Table Three below.

**Table 3.** Table consists of the revised initial programme theories in the form of CMO configurations. These are the initial programme theories that were agreed between the research team and the expert panel.

**CMO Configuration One - Connection** **Context:** A person who has experienced mental health challenges and as a result now attends a mental health service environment that enables/inhibits connection. **Mechanism:** Gaining/losing a sense of connection because of a person's point in their recovery at one set period in time. **Outcome:** Resulting in a person feeling/not feeling a sense of belonging, ease, being genuinely a part of something and finding one’s tribe in life. **CMO Configuration Two - Hope** **Context:** A person attending the mental health services is in an environment that allows them to either have hope or a loved one or staff holds hope whilst they work on their recovery. **Mechanism:** The service empowers/inhibits hope in a person in their recovery at a given moment in time. **Outcome:** Resulting in the person feeling hopeful for their ongoing recovery or hopeless regarding their future leading to behaviours that may or may not have a negative impact on a person’s physical and mental wellbeing. **CMO Configuration Three - Identity** **Context:** A person with lived/living experience of mental health challenges enters a mental health service where they discover/lose their identity. **Mechanism:** The person discovers/loses their identity due to their acceptance of lived reality at that moment in time. **Outcome:** Resulting in a person discovering/losing hope, gaining/losing a positive sense of self, of self-efficacy along with feeling stigmatised/destigmatised because of how they view their identity. **CMO Configuration Four – Meaning and Purpose** **Context:** A person who enters a mental health service for their mental health and wellbeing and whilst there, they discover/lose their sense of meaning and purpose in their lives. **Mechanism:** The person discovers/loses meaning and purpose in their lives on their recovery journey at that moment in time. **Outcome:** Leading to either a lack of/sense of self-worth, self-efficacy and a positive/negative sense of self and their placement within the world. **CMO Configuration Five - Empowerment** **Context:** A person experiencing mental health challenges enters a mental health service and whilst there, they become empowered/disempowered **Mechanism:** Due to their identity as a service user along with where they are in their recovery at that moment in time. **Outcome:** Leading to the person feeling/not feeling a sense of empowerment/disempowerment and self-efficacy. **CMO Configuration Six - CHIME** **Context:** A person who attends the mental health services enters an environment that is either conducive or not to CHIME. **Mechanism:** Due to their placement within this environment, the person discovers/loses connectedness, hope, identity, meaning and purpose and empowerment. **Outcome:** This results in a person feeling/not feeling a sense of hopefulness in their life which impacts their sense of belonging, ease, self, self-efficacy and self-worth, resulting in the person feeling stigmatised/destigmatised, which impacts their mental health recovery journey.

***Step two: searching for evidence.*** According to Pawson *et al*. ^34^, the next step of the realist review process will be to test these CMO configurations to see if the literature agrees and aligns with these hypothesised initial programme theories. In order to do this, the first step is to create a search strategy ^47^. Search strategy development should be an iterative process that results in a strategy to search the literature that is both sensitive and specific to the topic under investigation ^48^. Achieving this can be challenging and often requires several hours to properly construct ^49^. As such, obtaining the advice and expertise provided by a librarian is seen as crucial to the process ^50, 51^. In keeping with best practice, KW, an information specialist librarian from the Royal College of Surgeons in Ireland, was consulted in the search strategy development. As a result of the expertise of KWs, Table Four, Table Five and Table Six were constructed to contain the search terms that were devised to support the research team in gathering citations for this realist review from three databases: CINAHL, PubMed, and psychINFO. Grey literature will be gathered through a process of grey literature searching proposed by Godin *et al*. ^52^.

**Table 4.** Table describes the search strategy for the database: PubMed.

Table Four: Search Strategy for Pubmed			
*No.*	*Query*	*Last Run Via*	*Results*
#1	"Mental Health"[Mesh:NoExp] OR "Mental Disorders"[Mesh:NoExp] OR "mental health" OR "mental disorders" OR "psychiatric illness" OR "mental illness"	PubMed	6,58,228
#2	"chime" OR "c.h.i.m.e"	PubMed	610
#3	#1 AND #2	PubMed	79
#4	"Mental Health Recovery"[Mesh:NoExp] OR "Psychiatric Rehabilitation"[Mesh:NoExp] OR "mental health recovery" OR "psychological rehabilitation" OR "recovery" OR "personal recovery"	PubMed	6,71,015
#5	"Interpersonal Relations"[Mesh:NoExp] OR "Loneliness"[Mesh:NoExp] OR "Social Isolation"[Mesh:NoExp] OR "Social Participation"[Mesh:NoExp] OR "Community Participation"[Mesh:NoExp] OR "Social Alienation"[Mesh:NoExp] OR "connection" OR "belonging" OR "loneliness" OR "social isolation" OR "social participation"	PubMed	4,04,032
#6	"Hope"[Mesh:NoExp] OR "hope" OR "hopefulness"	PubMed	1,11,501
#7	"Individuality"[Mesh:NoExp] OR "Self Concept"[Mesh:NoExp] OR "identity" OR "self concept" OR "self worth" OR "sense of self"	PubMed	2,85,401
#8	"meaning" OR "purpose" OR "sense of purpose" OR "sense of meaning" OR "meaning making"	PubMed	17,37,065
#9	"Empowerment"[Mesh:NoExp] OR "Self Efficacy"[Mesh:NoExp] OR "empowerment" OR "self efficacy"	PubMed	78,289
#10	#3 AND #4	PubMed	60
#11	#3 AND #5	PubMed	7
#12	#3 AND #6	PubMed	41
#13	#3 AND #7	PubMed	39
#14	#3 AND #8	PubMed	40
#15	#3 AND #9	PubMed	40
#16	#9 OR #10 OR #11 OR #12 OR #13 OR #14 OR #15	PubMed	64

**Table 5.** Table describes the search strategy for the database: CINAHL.

Table Five: Search Strategy for CINAHL			
*No.*	*Query*	*Last Run Via*	*Results*
S1	(MH "Mental Health") OR "mental health" OR (MH "Mental Disorders") OR "mental disorders" OR "psychiatric illness" OR "mental illness"	E.Cinahl U	2,75,431
S2	"chime" OR "c.h.i.m.e"	E.Cinahl U	131
S3	S1 AND S2	E.Cinahl U	44
S4	(MH "Mental Health Recovery") OR "mental health recovery" OR (MH "Psychiatric Rehabilitation") OR "psychological rehabilitation" OR "recovery" OR "personal recovery"	E.Cinahl U	1,33,586
S5	"connection" OR "belonging" OR (MH "Interpersonal Relations") OR (MH "Loneliness") OR "loneliness" OR (MH "Social Isolation") OR "social isolation" OR (MH "Social Participation") OR "social participation" OR (MH "Community Participation") OR (MH "Social Alienation")	E.Cinahl U	1,35,585
S6	(MH "Hope") OR "hope" OR "hopefulness"	E.Cinahl U	29,184
S7	(MH "Individuality") OR "identity" OR (MH "Self Concept") OR "self concept" OR "self worth" OR "sense of self"	E.Cinahl U	94,517
S8	"meaning" OR "purpose" OR "sense of purpose" OR "sense of meaning" OR "meaning making"	E.Cinahl U	5,54,305
S9	(MH "Empowerment") OR "empowerment" OR (MH "Self Efficacy") OR "self efficacy"	E.Cinahl U	68,736
S10	S3 AND S4	E.Cinahl U	42
S11	S3 AND S5	E.Cinahl U	14
S12	S3 AND S6	E.Cinahl U	32
S13	S3 AND S7	E.Cinahl U	30
S14	S3 AND S8	E.Cinahl U	33
S15	S3 AND S9	E.Cinahl U	33
S16	S10 OR S11 OR S12 OR S13 OR S14 OR S15	E.Cinahl U	43

**Table 6.** Table describes the search strategy for the database: psychINFO.

Table Six: Search Strategy for psychINFO			
*No.*	*Query*	*Last Run Via*	*Results*
S1	DE "Mental Health" OR DE "Mental Disorders" OR "mental health" OR "mental disorders" OR "psychiatric illness" OR "mental illness"	E. APA PsyInfo	9,54,356
S2	"chime" OR "c.h.i.m.e"	E. APA PsyInfo	175
S3	S1 AND S2	E. APA PsyInfo	77
S4	"mental health recovery" OR "psychological rehabilitation" OR "recovery" OR "personal recovery"	E. APA PsyInfo	96,931
S5	DE "Loneliness" OR DE "Social Isolation" OR "connection" OR "belonging" OR "loneliness" OR "social isolation" OR "social participation"	E. APA PsyInfo	1,26,724
S6	DE "Hope" OR "hope" OR "hopefulness"	E. APA PsyInfo	54,010
S7	DE "Individuality" OR DE "Self-Concept" OR "identity" OR "self concept" OR "self worth" OR "sense of self"	E. APA PsyInfo	2,91,930
S8	"meaning" OR "purpose" OR "sense of purpose" OR "sense of meaning" OR "meaning making"	E. APA PsyInfo	5,10,927
S9	DE "Empowerment" OR DE "Self-Efficacy" OR "empowerment" OR "self efficacy"	E. APA PsyInfo	91,538
S10	S3 AND S4	E. APA PsyInfo	62
S11	S3 AND S5	E. APA PsyInfo	7
S12	S3 AND S6	E. APA PsyInfo	45
S13	S3 AND S7	E. APA PsyInfo	45
S14	S3 AND S8	E. APA PsyInfo	48
S15	S10 OR S11 OR S12 OR S13 OR S14	E. APA PsyInfo	63

The next step was to construct a pre-determined inclusion and exclusion criteria [ Table Seven]. A pre-determined inclusion/exclusion criteria is important, as it will be used to support the shortlisting of articles for this review. For this reason, each inclusion/exclusion criterion was selected for a reason. The rationale for this is now discussed below:

**Table 7.** Table describes the inclusion/exclusion criteria for this realist review.

Inclusion Criteria	Exclusion Criteria
Peer reviewed and grey literature based qualitative and mixed methods articles	Peer reviewed and grey literature based quantitative articles
	Literature reviews of any kind, opinion/perspective pieces, case studies, editorials, conference briefings
01 ^st^ January 2011-present	Articles published before 2011
Articles published in the English language	Articles published in languages that are not English
Mental Health [as defined by World Health Organisation ^64^] and Illness [Any mental illness as described by the International Classification of Diseases-11 ^65^ or Diagnostic and Statistical Manual of Mental Disorders 5-Text Revision ^66^	Addictions, Intellectual disabilities, physical health, dual diagnosis [of any kind]
Focuses explicitly on CHIME – connection, hope, identity, meaning and purpose and empowerment	Focuses on all other aspects of mental health recovery.

In the identification of appropriate resources for inclusion in this realist review, qualitative and mixed methods papers will be included, whereas quantitative papers will not. The rationale for this is that papers with qualitative data allow for theoretical depth to occur, which is not possible with other quantitative methodologies and positivist positionings. Literature reviews of any kind, including meta-synthesis, will be excluded because of the reviewers’ positionality towards the original raw data used in the original study ^1^. In other words, these review papers will be excluded as the reviewer is removed at least twice from the original raw data that was collected by the original study authors. As such, if reviews were included, it is possible that such reviews could misinterpret the original findings and make conclusions that are misleading due to the misinterpretation of the data presented by the original studies. Equally, the studies that report on the raw data included in such reviews may also be subject to bias or may not have been of sufficient quality, but yet included in such reviews due to, for example, a lack of papers identified for inclusion. As such, the findings of the review may not be accurate because of the inclusion of poorly conducted studies, which may impede the intended impact of the realist review. Grey literature will also be searched for relevant papers through a the process described by Godin and colleagues ^52^. Godin and colleagues approach involves a four step process: 1) searching grey literature databases, 2) customised Google search engines, 3) targeted websites and 4) consultation with contact experts ^52^. The articles to be collected will be from 2011 to the present [2025]. Articles published before 2011 will not be included as they would have been published before the literature review that created CHIME was published. Additionally, pre-2011 papers would have been captured by the original systematic review and narrative synthesis that constructed CHIME. Articles not published in English were excluded due to the inability of the review team in translating articles from other languages to English. Articles will be included only if they are relevant to mental health and illness. Articles discussing a dual diagnosis of any kind will be excluded because of the difficulties in distinguishing changes relating and not relating to mental health and illness alone. Finally, papers were also excluded if they spoke of an aspect of CHIME on their own and included if they spoke of all concepts of CHIME amalgamated together. Articles that examined other areas of mental health recovery were excluded from the review.

Once the inclusion/exclusion criteria are defined, the next step is to search the literature to determine whether these initial programme theories are credible or not. The planned search will use an exhaustive screening process that describes a systematic process whereby all citations that arise from applying the search strategy to databases will be included as part of round one screening. As part of the creation of this realist review protocol, the search strings were applied to the relevant databases, and all citations that arose were then placed into a citation software package Covidence to help manage the remainder of the screening process, which will be discussed in further detail as part of step three of the realist review process devised by Pawson *et al*. ^34^. Round one of the exhaustive screening process will be undertaken by both MJN and Information Specialist Librarian KW.

***Step three: select and appraise evidence.*** After round one of exhaustive searching concludes, a number of citations will have been found and added to the citation software package Covidence for the review team to review and narrow down further. Round two screening then occurs. Round two screening involves firstly eliminating any duplicates that may have been gathered as part of round one screening and then applying pre-determined inclusion/exclusion criteria [ Table Four] to the abstracts of the remainder of the citations gathered over the course of round one screening. Round two screening will be undertaken by MJN and ÉNS, who will screen 10% of the papers at this phase to ensure rigor and adherence to the pre-determined inclusion/exclusion criteria ^53^. Finally, round three screening will involve the application of a pre-determined inclusion/exclusion criteria [ Table Four] to the full text of citations included in this study. Again, this round was conducted by MJN with ÉNS, reviewing 10% of papers at this stage of searching also. If there are any disagreements regarding the inclusion or exclusion of a paper, then a third reviewer [JPB] will review the citation and make the ultimate decision regarding the paper’s inclusion or exclusion. Any citations left after the three rounds of screening were included in this realist review. As such, the process described above is similar to that of an original systematic review with a particular focus on thoroughness and transparency ^54^.

This realist review will appraise included studies for richness, relevance, and rigor in line with the realist methodology of Pawson *et al*. ^34^. According to Pawson *et al*., ^34^ relevance relates to whether the paper addresses the theory under investigation. In addition, rigor relates to how the research supports the conclusions drawn by the author of the paper being examined ^34^. Finally, richness refers to whether a paper has sufficient depth to meaningfully contribute to theory-building ^21^. Unlike systematic reviews that appraise for quality purposes ^55– 57^, in realist reviews, one appraises relevance as the development of theory ^21^. However, there is no agreed upon method for assessing relevance in realist reviews ^58^. As such, in this review, when appraising included studies, the following questions will be posed to the literature:

1. Does reading the full text confirm the paper context and feature CHIME explicitly?
2. Does the article provide concepts/data on outcomes?

***Step four: extract the data.*** Data will be gathered only from primary sources because of the issue of positionality when it comes to secondary sources, as noted by Norton ^1^. When it comes to primary sources, each citation will be examined thoroughly to identify the key aspects of each study, including the CMO configuration. Data collected from the citations that match these areas will be extracted by MJN into a data extraction form that will be developed by MJN prior to the commencement of this stage of Pawson and colleagues’ realist methodology. This data extraction document will be validated through consultation with the supervisory members of the review team (ÉNS, JPB, and TB). Once approved, this data extraction tool will test 10% of included studies using the below key aspects from each citation:

- Author[s], year and country of publication
- Aim of study
- Methodology utilised
- Sample and sample size
- Setting
- Study type – journal article, grey literature, dissertation etc.
- CHIME concept examined
- Context
- Mechanism
- Outcome

Once 10% of studies have their data extracted, ÉNS will review the data extraction tool and the data collected from extraction. If she is satisfied by the tool’s performance on 10% of included paper, the primary reviewer [MJN] will utilize this tool for the remainder of the papers to be extracted. The process of data extraction is iterative, meaning that the data extraction tool will be constantly refined over the course of the realist review process ^18^. It is also important to note that each paper may not contain a context, a mechanism or an outcome and as such, this can only be identified if the particular IPT is in itself identified. In other words, this protocol acknowledges that one cannot infer that something functions as a context or mechanism without first knowling the outcome it is relating to. In addition, rigor will be captured using the Crowe Critical Appraisal Tool ^59^. It was decided to utilise this tool as it has been utilised in another realist review within the health sciences space ^60^.

***Step five: analyse and synthesise data.*** The primary purpose of data synthesis is to determine what works, for whom, how, and under what circumstances, all in an attempt to refine the initial programme theories ^35^. In this way, the word synthesis in this context differs from the same word for systematic reviews, as in this process, the purpose of synthesis is to make progress in the explanation of initial programme theories into refined intial programme theories ^61^. In this realist review, two mechanisms are used to refine the initial programme theory. Firstly, summative content analysis will be conducted and will support the process of retroductive reasoning in order to develop the refined initial programme theories. Summative content analysis was devised by Hsieh and Shannon ^62^, and it involves an iterative process of identifying keywords based on the stated headings in stage four of Pawson and colleagues’ ^34^ realist methodology to create a set of refined intial programme theories. Once these refined intial programme theories are defined, the second process is a validation exercise where our subject experts appointed during stage one of the realist methodology will reconvene to approve the newly developed refined intial programme theories.

***Step six: ethics and dissemination.*** In line with similar realist reviews, ethical approval will not be required, as although we have an expert panel consisting of academics [ÉNS, JPB, TB] and expert panel members [MR, CB, DD], they have all been included in the authorship of this paper. In addition, no ethical approval is required, as no primary data collection will take place for the purpose of this review. They will also be included in the authorship of the realist review itself. Reporting bias will be contained through a process of reflexivity where the primary reviewer [MJN] will engage in journalling to support the process of retroductive reasoning whilst also acknowledging any reporting bias that may impede the fair development of the refined intial programme theories. The complete review will be presented as a second article and submitted for publication in a peer-reviewed journal. In addition, the results of this proposed realist review may also be presented through other means, including conferences and recovery workshops delivered by recovery college personnel. This will be made possible through the Recovery and Engagement Programme Manager in charge of the recovery education workstream of MHER.

## Conclusion

The present protocol provides a starting point for a comprehensive realist approach for a PhD project being conducted in order to understand the mechanisms that underpin the concept of personal recovery – in this case, CHIME ^8^. The first phase of this process is a realist review. This is useful because it allows for a theoretical examination of the various mechanisms that underpin CHIME, and will result in a theoretical interpretation of the underlying structures and mechanisms that support a person in reaching connection, hope, identity, meaning and purpose, and empowerment ^39^. To strengthen the realist review findings, the initial programme theories as well as the further developed refined intial programme theories were and will be examined by an expert panel who will then make recommendations for further refinement or agreement on the programme theories presented. To ensure that the papers selected for inclusion are relevant to the question posed, CHIME will need to be explicitly presented and referred to within the title, abstract, and full text of each included article in this realist review. This proposed realist review will add new knowledge to mental health recovery as it will either reinforce the initial programme theories created or provide new ones that can be investigated further in the next phase of the overarching study. As such, the findings of this proposed review will identify not only new avenues for exploration within CHIME, but also potential gaps in the knowledge base surrounding recovery that can be explored further. This proposed review is important, as it will also provide a unique method of exploring CHIME beyond the constraints of more traditional review methods. The results of this review will support work package two of the overarching PhD project which seeks to explore the inner mechanisms at play that allows a person to move from a state of unwellness towards recovery through CHIME.

## Data Availability

No data is associated with this article.
