# Supplementary material for: Understanding the Mechanisms that Operate within CHIME: A Realist Review Protocol
Source: HRB Open Res. 2025 Nov 17;8:94. Originally published 2025 Aug 26. [Version 2] doi: 10.12688/hrbopenres.14171.2 (PMC12648029; doi:10.12688/hrbopenres.14171.2)
Supplement: Peer review history

# Reviewer report: Reviewer response for version 2

**DOI:** 10.21956/hrbopenres.15730.r51644

**Published:** 2026-01-06

**Authors:** Sara Vilar-Lluch

**Affiliations:** 1 Cardiff University, Cardiff, UK

Overall very clear and informative. The writing needs some polishing, please see notes below. Occasionally, some glossing of concepts is needed (also noted below).

 Abstract:

 - CHIME is defined as “A concept that represents the key characteristics of recovery”: CHIME is not “a concept”, it’s a “framework”

 - Please rephrase the following sentence, the wording is not clear (i.e. clearly state what the question is): “allowing one to answer this review question by exploring how, why, and through what circumstances individuals reach recovery through CHIME.”

 - Is the following necessary, considering that this is meant to be indexing? > “The proposed review will be written up and submitted to a peer-reviewed journal”

Introduction

 p. 4: please ensure that a formal academic style is adopted throughout. For instance, in “However, till now, no study” > “till” should be “until”.

 p. 4: please revise wording “In answering this question, a realist review was deemed most appropriate” >> “to answer this question…” (please also note that a explicit research question is not provided; it is formulated as an aim).

 p. 4: please note that the last paragraph of the introduction starts and ends in the same manner (stating the aims of the review). Please avoid repetitions.

Methods

 p. 4: please revise wording (repetition of verb “are”): “Realist reviews are, by their very

 nature, are cyclical and iterative in their approach”

 Table 1 (p, 6). All the “if… then…” statements start with “If a person with lived experience of mental health challenges” (or similar) except point 4 (“If a person who uses community mental health services”). Unless there is a reason for this change (in which case should be explained), please unify.

 p. 6: the relevance of physical proximity is not clear, if the meetings were online, please amend as necessary: “Due to the proximity of all parties to one another in the country, the expert panel convened online via MS Teams and lasted approximately an hour and a half”

 p. 6: please state the sixth element at the end of this sentence: “After the meeting, the original CMOs were amended, and a sixth CMO was added based on the discussion.”

 p. 6: please briefly explain the process alluded to after this sentence (or indicate which section of the paper provides this information if this is the case): “Grey literature will be gathered through a process of grey literature searching proposed by Godin *et al*.” This is then repeated at p. 8 (and the process is briefly explained); just refer there – or move the explanation to the methods, together with the other explanations.

 p. 6: this sentence appears redundant, please amend or simply delete, and introduce the rationale directly: “For this reason, each inclusion/exclusion criterion was selected for a reason.”

 p. 6: please unify verbal tenses. The methods were reported in past tense, but then there is a shift to future tense: “In the identification of appropriate resources for inclusion in this realist review, qualitative and mixed methods papers will be included, whereas quantitative papers will not”. Please unify.

 p. 8: please simply the following three sentences (they repeat the same information in different words and appear repetitive): “Literature reviews of any kind, including meta-synthesis, will be excluded because of the reviewers’ positionality towards the original raw data used in the original study1. In other words, these review papers will be excluded as the reviewer is removed at least twice from the original raw data that was collected by the original study authors. As such, if reviews were included, it is possible that such reviews could misinterpret the original findings and make conclusions that are misleading due to the misinterpretation of the data presented by the original studies.”

 p. 8: please rephrase this sentence, it appears odd: “Articles published before 2011 will not be included as they would have been published before the literature review that created CHIME was published.”

 p. 8: please unify tenses (here one is in past, and the following is in the future): “Articles not published in English were excluded due to the inability of the review team in translating articles from other languages to English. Articles will be included only if they are relevant to mental health and illness.” (same in the first paragraph of p. 9)

 p. 10: first paragraph. As before, there is inconsistency of tenses; the preceding sentence to the one copied is in future tense, this one in past (and the one that follows in future): “Again, this round was conducted by MJN with ÉNS, reviewing 10% of papers at this stage of searching also.” I understand that the paper reports a work in progress, but if this is meant to be a protocol, tenses need unification, otherwise it is confusing.

 p. 12: “rigor will be captured using the Crowe Critical Appraisal Tool” > please provide a brief gloss of this tool, it is not explained.

Is the study design appropriate for the research question?

Yes

Is the rationale for, and objectives of, the study clearly described?

Yes

Are sufficient details of the methods provided to allow replication by others?

Yes

Are the datasets clearly presented in a useable and accessible format?

Not applicable

Reviewer Expertise:

Health communication, recovery, applied linguistics

I confirm that I have read this submission and believe that I have an appropriate level of expertise to confirm that it is of an acceptable scientific standard.

# Reviewer report: Reviewer response for version 2

**DOI:** 10.21956/hrbopenres.15730.r51531

**Published:** 2025-12-25

**Authors:** Dr. Jutharat Thongsalab

**Affiliations:** 1 Boromarajonani College of Nursing Surin, Mueang Surin District, Surin, Thailand

Thank you for the revised version of this protocol. The authors have addressed several earlier comments. These changes have clearly enhanced the clarity and structure of the protocol. Some methodological aspects (e.g., proportion of double-screening, inter-rater agreement, and the decision to exclude quantitative studies) could be further strengthened, but these issues do not fundamentally compromise the coherence or feasibility of the proposed realist review. Furthermore, I recommend accepting this protocol.

Is the study design appropriate for the research question?

Yes

Is the rationale for, and objectives of, the study clearly described?

Partly

Are sufficient details of the methods provided to allow replication by others?

Partly

Are the datasets clearly presented in a useable and accessible format?

Partly

Reviewer Expertise:

NA

I confirm that I have read this submission and believe that I have an appropriate level of expertise to confirm that it is of an acceptable scientific standard.

# Reviewer report: Reviewer response for version 2

**DOI:** 10.21956/hrbopenres.15730.r51529

**Published:** 2025-11-26

**Authors:** Chalotte Heinsvig Poulsen

**Affiliations:** 1 Copenhagen Research Unit for Recovery, Copenhagen, Denmark; 2 Mental Health Center Glostrup, Centre for Applied Research in Mental Health, Mental Health Services, Capital region of denmark, Copenhagen, Denmark

I can read that you have accommodated my feedback regarding transparency and the positioning of the expert panel and other suggestions. I have no further comments.

Is the study design appropriate for the research question?

Yes

Is the rationale for, and objectives of, the study clearly described?

Yes

Are sufficient details of the methods provided to allow replication by others?

Partly

Are the datasets clearly presented in a useable and accessible format?

Not applicable

Reviewer Expertise:

Post doc researcher; Co-production; Peer support; Recovery-oriented research; Development of program theory; Critical realism; Proces evaluation; Randomized controlled trials supplemented by qualitative research about mechanisms of change and context.

I confirm that I have read this submission and believe that I have an appropriate level of expertise to confirm that it is of an acceptable scientific standard.

# Reviewer report: Reviewer response for version 2

**DOI:** 10.21956/hrbopenres.15730.r51530

**Published:** 2025-11-25

**Authors:** Geoff Wong

**Affiliations:** 1 University of Oxford, Oxford, UK

Thanks for taking the time to read and consider my comments.

 As none of the comments I made were major and had to be addressed, I hope you found them helpful.

 Good luck with your review.

Is the study design appropriate for the research question?

Yes

Is the rationale for, and objectives of, the study clearly described?

Yes

Are sufficient details of the methods provided to allow replication by others?

Partly

Are the datasets clearly presented in a useable and accessible format?

Not applicable

Reviewer Expertise:

Realist review / synthesis and realist evaluation.

I confirm that I have read this submission and believe that I have an appropriate level of expertise to confirm that it is of an acceptable scientific standard.

# Reviewer report: Reviewer response for version 1

**DOI:** 10.21956/hrbopenres.15583.r50797

**Published:** 2025-11-11

**Authors:** Chalotte Heinsvig Poulsen

**Affiliations:** 1 Copenhagen Research Unit for Recovery, Copenhagen, Denmark; 2 Mental Health Center Glostrup, Centre for Applied Research in Mental Health, Mental Health Services, Capital region of denmark, Copenhagen, Denmark

Thank you for allowing me to read and peer review this thorough and well-written realist review protocol. The aim is clearly stated as a realist systematic review of qualitative studies focusing on the underlying mechanisms that facilitate the transition from mental distress to wellbeing and personal recovery, as described in the CHIME framework. The methodology is comprehensive, following criteria for realist reviews with a detailed and transparent account of programme theory development, research questions, search strategy, article selection, and reporting. The approach is easy to understand and reproducible for other researchers, and the process is effectively illustrated with the included figures and tables.

 The authors could benefit from further clarifying the following points:

 1) Describe the positions of the author group - are anyone working from a lived experience of mental health recovery perspective? -  and/or caregiver perspective?

 2) More information about PPI research - the service users in the expert panel - who are they, how were they recruited? How is diversity, equal power sharing, safe and secure user involvement ensured?

 3) To which degree was the expert panel involved? - was it user-involvement, co-production or co-creation?

 4) How will the program theory iteratively be refined based on emerging data?

 5) Do the authors have a plan for managing potential heterogeneity in study contexts and populations, and how will this be addressed in the synthesis?

 6) Discussion of potential limitations inherent to realist systematic reviews compared to other types of reviews and how these will be mitigated.

 7) Be aware that if the CHIME agronym should be explicitly presented and referred to within the title, abstract and full text - will exclude papers for example the attached paper by Egmose et al. 2024 - exploring the mechanisms of change in a group-based peer support intervention co-produced around the CHIME framework.

Is the study design appropriate for the research question?

Yes

Is the rationale for, and objectives of, the study clearly described?

Yes

Are sufficient details of the methods provided to allow replication by others?

Partly

Are the datasets clearly presented in a useable and accessible format?

Not applicable

Reviewer Expertise:

Post doc researcher; Co-production; Peer support; Recovery-oriented research; Development of program theory; Critical realism; Proces evaluation; Randomized controlled trials supplemented by qualitative research about mechanisms of change and context.

I confirm that I have read this submission and believe that I have an appropriate level of expertise to confirm that it is of an acceptable scientific standard, however I have significant reservations, as outlined above.

## References

[1] : The ‘Paths to everyday life’ (PEER) trial – a qualitative study of mechanisms of change from the perspectives of individuals with mental health difficulties participating in peer support groups led by volunteer peers. *BMC Psychiatry* .2024;24(1) : 10.1186/s12888-024-05992-w 10.1186/s12888-024-05992-w 39138435 PMC11321162

# Response

**Published:** 2025-11-11

**Authors:** Michael John Norton

- **Thank you for allowing me to read and peer review this thorough and well-written realist review protocol. The aim is clearly stated as a realist systematic review of qualitative studies focusing on the underlying mechanisms that facilitate the transition from mental distress to wellbeing and personal recovery, as described in the CHIME framework. The methodology is comprehensive, following criteria for realist reviews with a detailed and transparent account of programme theory development, research questions, search strategy, article selection, and reporting. The approach is easy to understand and reproducible for other researchers, and the process is effectively illustrated with the included figures and tables.**
- **Response: **Thank you for your kind comment. We hope that the amendments made to this paper as a result of your feedback and the feedback of the other peer reviewers enhances the protocol further. 
- **The authors could benefit from further clarifying the following points:**
** 1) Describe the positions of the author group - are anyone working from a lived experience of mental health recovery perspective? -  and/or caregiver perspective?**
- **Response: **Thank you for your comment. As per your feedback and the feedback of another reviewer, the positions of the author group are noted as part of stage one of the iterative process of realist reviews.
- **2) More information about PPI research - the service users in the expert panel - who are they, how were they recruited? How is diversity, equal power sharing, safe and secure user involvement ensured?**
- **Response: **Thank you for this comment. PPI is central to this proposed research. The principal investigator is a service user himself along with two of the expert panel members. This ensures that the views of service users are central to the creation of this protocol and the subsequent review. As part of the realist review process, the refined IPTs will be presented to the expert panel for approval once data collection is completed. In answering point 1 above, we have already identified the expert panel personal. They were recruited from the principal investigators contacts through his work with the HSE Office of Mental Health Engagement and Recovery – an office that promotes recovery, co-production and service user involvement within mental health service provision. Finally, equal power sharing is ensured as all members of the expert panel have been involved in service user involvement and co-production for a number of years and fully understands the nuances of these processes not just in service provision but also in research. 
- **3) To which degree was the expert panel involved? - was it user-involvement, co-production or co-creation?**
- **Response: **Thank you for this comment. The expert panel were involved in the process through the co-production of the initial IPTs and will be involved again in the refinement of the IPTs through co-production. 
- **4) How will the program theory iteratively be refined based on emerging data?**
- **Response: **Thank you for this comment. The protocol outlines the process of how the papers were gathered and how the papers will be reviewed. Part of this process will be drawing out the context, mechanisms and outcomes of all the included papers. These will then be put together by aligning the new IPTs to the various elements of CHIME to see what refined IPTs relate to each concept and which concepts intertwine with each other. Additionally, these refined IPTs will be compared to the original IPTs that were co-produced with the expert panel to identify if what we found matches with these original IPTs. To further support this. The IPTs will refer to micro [personal], meso [service] and macro [system] factors and as such, these IPTs will be placed along this continuum with a diagram visually depicting this. This will then be brought to the expert panel for approval and once approved, write up of the realist review will commence. 
- **5) Do the authors have a plan for managing potential heterogeneity in study contexts and populations, and how will this be addressed in the synthesis?**
- **Response: **Thank you for this comment. This query is answered through point 4 above. We do anticipate heterogeneity in context and populations but this will be demonstrated through aligning the IPTs to a micro, meso and macro perspective where contexts in terms of individual, service or system will be demonstrated. This heterogeneity will also be visually demonstrated. 
- **6) Discussion of potential limitations inherent to realist systematic reviews compared to other types of reviews and how these will be mitigated.**
- **Response: **Thank you for this comment. This review differs from systematic reviews and scoping reviews as it is theory heavy, and the focus is on looking for potential mechanisms rather than examining potential depth and breadth of the literature base. This does raise questions of potential bias but this is mitigated because the focus is on developing mechanisms rather than elimination of bias. 
- **7) Be aware that if the CHIME agronym should be explicitly presented and referred to within the title, abstract and full text - will exclude papers for example the attached paper by Egmose et al. 2024 - exploring the mechanisms of change in a group-based peer support intervention co-produced around the CHIME framework.**
- **Response: **Thank you for this comment. We are aware that focusing on CHIME in these areas will exclude paper. However, as noted in point 6 the focus is on finding mechanisms, not on ensuring that the breadth/depth of literature is achieved. 

# Reviewer report: Reviewer response for version 1

**DOI:** 10.21956/hrbopenres.15583.r50799

**Published:** 2025-10-27

**Authors:** Geoff Wong

**Affiliations:** 1 University of Oxford, Oxford, UK

Overall comments:

 The manuscript is clearly written and a coherent explanation is provided for the use of a realist review approach.

 Major comments:

 None.

 Minor comments:

 As this is a realist review, in your manuscript you may want to consider only using the word "mechanism" when you specifically mean it in a realist sense of the word - i.e. as a hidden, context-sensitive causal force.

  When reference is first made about the RAMESES I guidelines, there is mention of these standards being used to "guide the review itself and not its execution." I'm not quite sure what this means. The references 30 and 31 refer to the RAMESES publication standards for realist syntheses and so should be used to guide the reporting of realist syntheses (or reviews - the terms are synonymous). If guidance is needed for the conduct of realist syntheses, the the reference used should be:

 Wong G, Greenhalgh T, Westhorp G, Pawson R. Development of methodological guidance, publication standards and training materials for realist and meta-narrative reviews: the RAMESES (Realist And Meta-narrative Evidence Syntheses – Evolving Standards) project. *Health Soc Care Deliv Res* 2014;2(30). [Reference 1]

 When programme theory is mentioned in step 1, it is worth remembering that a programme theory is both a theory of change, and a theory of implementation / action. See either the RAMESES training materials for realist syntheses:https://www.ramesesproject.org/pgm-download_media.php?name=Realist_reviews_training_materials.pdf

 or

 here: https://www.ramesesproject.org/pgm-download_media.php?name=RAMESES_II_Theory_in_realist_evaluation.pdf

 The steps proposed for the realist review make sense.

 In step 1, a reasonable process has been undertaken to develop the CMO configurations that will populate the initial programme theory. These initial CMO configurations seem to me a reasonable first stab at providing causal explanations.

 Based on the details provided, the searches seem sensible and likely to find relevant data. Explanations are provided for the exclusion criteria what will be used. These on the whole make sense, though, you may want to reconsider excluding quantitative papers as these may provide 'nuggets' of relevant data that enable you to achieve a better degree of consilience.

 I am no content expert, but if the seminal papers for CHIME were published in 2011, there may be relevant papers published just before 2011. Hence, if you have not done so, it  may be worth checking if setting the cut off 2 to 3 years earlier might yield any relevant papers.

 Whilst I can understand t-your rationale for excluding reviews, do consider citation tracking from the reviews - as this may be a means of identifying further relevant documents.

 In step 3, the selection and appraisal process makes sense. The three questions you propose seem a bit like overkill as relevance can be considered as whether a document has data that can contribute to realist programme theory in some way (e.g. to inform CMOCs, their relationships to each other or an aspect of the programme theory).

 You have not mentioned appraisal of rigour. On this issue you may find the second half of this book chapter of use: Data gathering for realist reviews: Looking for needles in haystacks. Wong G. In: Emmel N, Greenhalgh J, Manzano A, Monaghan M, Dalkin S, editors. Doing Realist Research. London: Sage, 2018 [Reference 2]

 In Step 4, I am not sure that it is ever as clean cut as to claim "In general, the introduction of citations will identify the context, the results of these same citations will identify the outcome, and the discussion of the text will help identify the mechanism.". You are likely to find that relevant data may come from any part of a document. Hence some flexibility in where you draw your data from will likely be needed.

 In your data extraction tool, you may not be able to find data that is specifically about context, mechanism or outcome. This is because such data on each of these concepts may not be present in one single document. And, it is challenging to be able to claim that something is functioning as context or mechanism, unless you can identify in which CMOC it is operating in. In other words, you can only infer that something is functioning as a context or mechanism if you know what the outcome it is related to.

 With regard to the use of the Crowe Critical Appraisal Tool, I presume you are planning to use it to appraise rigour and not relevance? If so, do consider reading the  Data gathering for realist reviews book chapter that I have mentioned above, as following the process outlined in that book chapter may save you a lot of time and effort.

 In step 5 consider revising this sentence " all in an attempt to refine the initial programme theories into middle-range theories", because middle-range theory is not a type of theory, but more a description of the level of abstraction of a theory. See: https://www.ramesesproject.org/pgm-download_media.php?name=RAMESES_II_Theory_in_realist_evaluation.pdf

 In this step, you have not provided much detail on how you will develop the causal explanations (i.e. CMOCs) and programme theory. Whilst this is not necessarily needed in a protocol, you should ensure you provide such details for your PhD. There is no one set process you have to use to make sense of data, mainly because we all 'think' differently. But any process you use should be coherent, clearly explained and also used systematically.

Is the study design appropriate for the research question?

Yes

Is the rationale for, and objectives of, the study clearly described?

Yes

Are sufficient details of the methods provided to allow replication by others?

Partly

Are the datasets clearly presented in a useable and accessible format?

Not applicable

Reviewer Expertise:

Realist review / synthesis and realist evaluation.

I confirm that I have read this submission and believe that I have an appropriate level of expertise to confirm that it is of an acceptable scientific standard, however I have significant reservations, as outlined above.

## References

[1] : Development of methodological guidance, publication standards and training materials for realist and meta-narrative reviews: the RAMESES (Realist And Meta-narrative Evidence Syntheses – Evolving Standards) project. *Health Services and Delivery Research* .2014;2(30) : 10.3310/hsdr02300 1-252 10.3310/hsdr02300 25642521

[2] : Data Gathering in Realist Reviews: Looking for needles in haystacks.2018; 10.4135/9781526451729.n9 131-146 10.4135/9781526451729.n9

# Response

**Published:** 2025-11-08

**Authors:** Michael John Norton

- **The manuscript is clearly written and a coherent explanation is provided for the use of a realist review approach.**
- **Response: **Thank you for this comment. We hope that the amendments made as per your suggestions below will improve the overall usefulness of the protocol for our review.
- **As this is a realist review, in your manuscript you may want to consider only using the word "mechanism" when you specifically mean it in a realist sense of the word - i.e. as a hidden, context-sensitive causal force.**
- **Response: **Thank you for your comment. We have reread the protocol and have only used mechanisms when referring to a hidden, context-sensitive causal force as suggested.
- **When reference is first made about the RAMESES I guidelines, there is mention of these standards being used to "guide the review itself and not its execution." I'm not quite sure what this means. The references 30 and 31 refer to the RAMESES publication standards for realist syntheses and so should be used to guide the reporting of realist syntheses (or reviews - the terms are synonymous). If guidance is needed for the conduct of realist syntheses, the reference used should be:**
** Wong G, Greenhalgh T, Westhorp G, Pawson R. Development of methodological guidance, publication standards and training materials for realist and meta-narrative reviews: the RAMESES (Realist And Meta-narrative Evidence Syntheses – Evolving Standards) project. *Health Soc Care Deliv Res* 2014;2(30).**
- **Response: **Thank you for your comment. We have reviewed the text in question and have amended same accordingly. We have also inserted your reference to strengthen the claims made.
- **When programme theory is mentioned in step 1, it is worth remembering that a programme theory is both a theory of change, and a theory of implementation / action. See either the RAMESES training materials for realist syntheses: WEBSITE.pdf **
- **Response: **Thank you for this comment. We have added a sentence to acknowledge this in the revised protocol.
- **The steps proposed for the realist review make sense.**
- **Response: **Thank you for this comment.
- **In step 1, a reasonable process has been undertaken to develop the CMO configurations that will populate the initial programme theory. These initial CMO configurations seem to me a reasonable first stab at providing causal explanations.**
- **Response: **Thank you for this comment.
- **Based on the details provided, the searches seem sensible and likely to find relevant data. Explanations are provided for the exclusion criteria what will be used. These on the whole make sense, though, you may want to reconsider excluding quantitative papers as these may provide 'nuggets' of relevant data that enable you to achieve a better degree of consilience.**
- **Response: **Thank you for your comment. We have excluded quantitative studies as personal recovery is a subjective process that cannot be adequately captured through positivist, quantitative means. Additionally, quanitative research lacks contextual depth and given that realist reviews aim to answer questions relating to why, how and under what circumstances a mechanism occurs, it was deemed inappropriate to include quanitative studies.
- **I am no content expert, but if the seminal papers for CHIME were published in 2011, there may be relevant papers published just before 2011. Hence, if you have not done so, it  may be worth checking if setting the cut off 2 to 3 years earlier might yield any relevant papers.**
- **Response: **Thank you for also raising this comment. We restricted the inclusion/exclusion criteria to post 2011 studies for two reasons. Firstly, CHIME was constructed in a 2011 paper from Mary Leamy and colleagues in the UK and as a result, this review is looking for papers that include all elements of CHIME. Secondly, we also excluded pre-2011 studies as these would have been captured by the original systematic review method conducted by Leamy et al. when they carried out their review. As such, it was deemed unnecessary to include pre-2011 research studies.
- **Whilst I can understand your rationale for excluding reviews, do consider citation tracking from the reviews - as this may be a means of identifying further relevant documents.**
- **Response: **Thank you for your comment. We are unable to conduct citation chaining for this review as we are now presently finalising extraction and commencing write up of the actual realist review. However, we will endeavour to include in our methodology that citation chaining was recommended but was unable to be carried out due to the phase of the review where reviewers were at when your peer review comment was made.
- **In step 3, the selection and appraisal process makes sense. The three questions you propose seem a bit like overkill as relevance can be considered as whether a document has data that can contribute to realist programme theory in some way (e.g. to inform CMOCs, their relationships to each other or an aspect of the programme theory).**
- **Response: **Thank you for this comment. We have reviewed the text based on your feedback and revised it accordingly.
- **You have not mentioned appraisal of rigour. On this issue you may find the second half of this book chapter of use: Data gathering for realist reviews: Looking for needles in haystacks. Wong G. In: Emmel N, Greenhalgh J, Manzano A, Monaghan M, Dalkin S, editors. Doing Realist Research. London: Sage, 2018**
- **Response: **Thank you for this comment. We have addressed appraisal of rigor through step 4 of the realist review process.
- **In Step 4, I am not sure that it is ever as clean cut as to claim "In general, the introduction of citations will identify the context, the results of these same citations will identify the outcome, and the discussion of the text will help identify the mechanism.". You are likely to find that relevant data may come from any part of a document. Hence some flexibility in where you draw your data from will likely be needed.**
- **Response: **Thank you for this comment. We have reviewed the piece of text highlighted and decided to remove the highlighted text.
- **In your data extraction tool, you may not be able to find data that is specifically about context, mechanism or outcome. This is because such data on each of these concepts may not be present in one single document. And, it is challenging to be able to claim that something is functioning as context or mechanism, unless you can identify in which CMOC it is operating in. In other words, you can only infer that something is functioning as a context or mechanism if you know what the outcome it is related to.**
- **Response: **Thank you for this comment. We have made note of this and included it under stage 4 of the realist review process within the discussions around the data extraction tool.
- **With regard to the use of the Crowe Critical Appraisal Tool, I presume you are planning to use it to appraise rigour and not relevance? If so, do consider reading the  Data gathering for realist reviews book chapter that I have mentioned above, as following the process outlined in that book chapter may save you a lot of time and effort.**
- **Response: **Thank you for this comment. Yes, we plan to use it to appraise rigour and not relevance as previously stated. Thank you for your suggested reading, however, we are unable to implement it as we are already finalising data extraction and appraisal for the realist review itself at this time.
- **In step 5 consider revising this sentence " all in an attempt to refine the initial programme theories into middle-range theories", because middle-range theory is not a type of theory, but more a description of the level of abstraction of a theory. See: WEBSITE.pdf**
- **Response: **Thank you for this comment. We have revised this sentence based on your suggestion and have reviewed the remainder of the document to remove any reference to middle-ranged theory.
- **In this step, you have not provided much detail on how you will develop the causal explanations (i.e. CMOCs) and programme theory. Whilst this is not necessarily needed in a protocol, you should ensure you provide such details for your PhD. There is no one set process you have to use to make sense of data, mainly because we all 'think' differently. But any process you use should be coherent, clearly explained and also used systematically.**
- **Response: **Thank you for this comment. We will keep this in mind for the actual realist review paper as suggested.

# Reviewer report: Reviewer response for version 1

**DOI:** 10.21956/hrbopenres.15583.r49952

**Published:** 2025-09-30

**Authors:** Dr. Jutharat Thongsalab

**Affiliations:** 1 Boromarajonani College of Nursing Surin, Mueang Surin District, Surin, Thailand

Thank you for the opportunity to review this interesting and timely protocol. The topic is important and relevant for advancing recovery-oriented mental health research. The paper is well-structured and demonstrates strong potential. I provide the following comments to strengthen clarity, methodological rigor, and practical contribution.

 - I recommended that the author support the gap statement by systematically highlighting what CHIME research has achieved (measurement, validation, intervention use) and what remains unexplored (mechanisms, context-specific functioning).

 - The introduction part lacks a sharp demonstration of the evidence gap, a critical comparison with other review types, and a clear link to practice implications. Strengthening these aspects will make justification more persuasive.

**- **The methods section is full and clear in structure but would benefit from greater rigor in evidence appraisal, inclusion criteria, extraction reliability, and analytic strategy. With these refinements, the review will be more credible and reproducible.

 - Step I, the author should clarify procedures for iterative refinement, include safeguards against confirmation bias, and discuss diversity/independence of the advisory panel.

 - Step II, Exclusion of quantitative studies is restrictive; yet some quantitative work (e.g., mediation, SEM, longitudinal studies) could inform mechanisms. Restricting to post-2011 excludes potentially relevant pre-CHIME conceptualizations that could enrich theory building. Reconsider exclusion of quantitative studies, broaden the grey literature strategy, and justify more clearly the 2011 cutoff.

 - Step III, Screening and appraisal are described (three rounds; Covidence; 10% double-screening). Appraisal using “relevance, rigor, richness” is conceptually valid but subjective; no plan for handling disagreements or measuring inter-rater agreement. Therefore, increase the proportion of double-screening, consider structured appraisal checklists (e.g., CASP, RAMESES appraisal tool), and report inter-rater reliability (e.g., Cohen’s κ).

 - Step IV doesn’t specify whether pilot testing of the extraction form will be done. Reliance on a single reviewer (MJN) for extraction, with supervisory oversight, carries a risk of bias.

 - Step V, the process described could produce descriptive categories rather than true realist synthesis. Explicitly describe the use of retroductive reasoning, provide examples of how initial CMOs will evolve into middle-range theories, and consider external validation beyond the author panel.

 - Step VI, add transparency on how independence of critique will be preserved, and describe dissemination beyond academic journals (e.g., co-produced outputs for service users).

 - Figure 2 uses “Inductive Reflexive Thematic Analysis” under synthesis. This terminology is more common in qualitative research, but realist reviews emphasize retroductive reasoning and CMO refinement.

 - The current wording may mislead readers about analytic methods. This figure presents the steps as linear, but realist reviews are inherently iterative (moving back and forth between theory building, searching, and refinement). This could misrepresent the cyclical nature of realist reviews. Some steps (e.g., “Upload onto NVivo for Analysis”) are tool-specific, which may not apply universally and make the figure feel like a workflow chart rather than a generalizable method. Meanwhile, other important elements (e.g., stakeholder engagement, retroductive reasoning, theory testing vs. refinement) are missing. Advisory panel engagement should appear not just at the start, but also during synthesis/validation.

 - Table 1 provides a useful but overly simplistic starting point for realist program theory development. It will benefit if you clarify distinctiveness by ensuring each CHIME domain’s If…The statement highlights unique mechanisms. Add one illustrative case or a brief exemplary per domain to ground the theory.

 - Table 2 shows the evolution into draft CMOs. I recommend improvement for it.  The context should add diversity in settings (community vs. inpatient), roles (peer vs. clinician support), and cultural/systemic factors. Mechanisms should frame them as resources + reasoning (what is offered + how it is interpreted), e.g., “Access to peer groups (resource) triggers recognition of shared experience (reasoning)”. Outcomes should be more diverse by going beyond re-stating mechanisms, e.g., linking hope, such as increased engagement, reduced suicidality, and improved functioning.

 - Table 3 remains too generic, tautological, and individual-focused. With richer contextualization, sharper mechanism articulation, and more concrete outcomes, it could provide a robust foundation for theory testing in the review.

 - Table 4-6, Search strings like “Mental Health” OR “Mental Disorders” yield millions of hits (e.g., 6.5M in PubMed). This risks irrelevance and screening overload. It might indicate poor precision in early steps. Lack of Grey Literature Strategy in These Tables. Excluding pre-2011 studies may miss early CHIME conceptual development or precursors. Needs a stronger justification than “CHIME was published in 2011.

 - Table 7, please reconsider including quantitative studies that explore mechanisms (e.g., mediation, regression, factor analysis). Provide a rationale for excluding dual diagnosis/addiction populations or consider including them with sensitivity analysis.

Is the study design appropriate for the research question?

Yes

Is the rationale for, and objectives of, the study clearly described?

Partly

Are sufficient details of the methods provided to allow replication by others?

Partly

Are the datasets clearly presented in a useable and accessible format?

Partly

Reviewer Expertise:

Psychiatric and Mental Health Nursing; Recovery-Oriented Care; Nursing Research Methods; Systematic and Review Methodology.

I confirm that I have read this submission and believe that I have an appropriate level of expertise to confirm that it is of an acceptable scientific standard, however I have significant reservations, as outlined above.

# Response

**Published:** 2025-11-08

**Authors:** Michael John Norton

- **Thank you for the opportunity to review this interesting and timely protocol. The topic is important and relevant for advancing recovery-oriented mental health research. The paper is well-structured and demonstrates strong potential. **
- **Response:** Thank you for your comment. We hope that the amendments made to this paper will strengthen it further. 
- **I provide the following comments to strengthen clarity, methodological rigor, and practical contribution.**
- **Response: **Thank you for this. We hope that the amendments the authorship team have accepted will have strengthened the paper further.
- **I recommended that the author support the gap statement by systematically highlighting what CHIME research has achieved (measurement, validation, intervention use) and what remains unexplored (mechanisms, context-specific functioning).**
- **Response: **Thank you for this comment. We have adjusted the rationale for realist review section to incorporate this comment.
- **The introduction part lacks a sharp demonstration of the evidence gap, a critical comparison with other review types, and a clear link to practice implications. Strengthening these aspects will make justification more persuasive.**
- **Response: **Thank you for this comment. The introduction has been sharpened to include the evidence gap and comparison with other review types along with a clearer link been made to practice implications.
- **The methods section is full and clear in structure but would benefit from greater rigor in evidence appraisal, inclusion criteria, extraction reliability, and analytic strategy. With these refinements, the review will be more credible and reproducible.**
- **Response: **Thank you for this comment. We are not trying to produce a systematic review which would rely on reproducibility. We are instead utilising a realist review methodology to uncover mechanisms. Although the search was rigorous, it does not need to be of the same standard as a systematic review where your concerns are of extreme importance. The evidence appraisal method has been taken and adopted from previous realist reviews which utilise this method. The inclusion criteria are robust given the review method. We believe you have commented on some of the other aspects of the inclusion criteria below, which we will address then. The extraction process follows a realist method and the analytic process is suitable, given the review type.
- **Step I, the author should clarify procedures for iterative refinement, include safeguards against confirmation bias, and discuss diversity/independence of the advisory panel.**
- **Response: **Thank you for your comment. Given the review type, safeguards against confirmation bias is not necessary. In regard to the diversity and independence of the advisory panel, the text now discusses these in step 1 of the realist review process.
- **Step II, Exclusion of quantitative studies is restrictive; yet some quantitative work (e.g., mediation, SEM, longitudinal studies) could inform mechanisms.**
- **Response: **Thank you for raising this comment. A conscious decision was made to exclude quantitative studies as personal recovery is a subjective experience that cannot be captured adequately through positivist quantitative means. In addition, quantitative research lacks contextual depth and given that realist reviews aim to demonstrate how, why and under what circumstances a mechanism operates, the inclusion of quanitative studies was deemed inappropriate.
- **Restricting to post-2011 excludes potentially relevant pre-CHIME conceptualizations that could enrich theory building.** 
- **Response: **Thank you for also raising this comment. We restricted the inclusion/exclusion criteria to post 2011 studies for two reasons. Firstly, CHIME was constructed in a 2011 paper from Mary Leamy and colleagues in the UK and as a result, this review is looking for papers that include all elements of CHIME. Secondly, we also excluded pre-2011 studies as these would have been captured by the original systematic review method conducted by Leamy et al. when they carried out their review. As such, it was deemed unnecessary to include pre-2011 research studies.
- **Reconsider exclusion of quantitative studies, broaden the grey literature strategy, and justify more clearly the 2011 cutoff.**
- **Response: **Thank you for this comment. The rationale for excluding quantitative studies have been mentioned above. A couple of sentences has been added to describe the grey literature search mechanisms. The justification for the 2011 cutoff has been discussed above.
- **Step III, Screening and appraisal are described (three rounds; Covidence; 10% double-screening). Appraisal using “relevance, rigor, richness” is conceptually valid but subjective; no plan for handling disagreements or measuring inter-rater agreement. Therefore, increase the proportion of double-screening, consider structured appraisal checklists (e.g., CASP, RAMESES appraisal tool), and report inter-rater reliability (e.g., Cohen’s κ).**
- **Response: **Thank you for this comment. Given the theory focus of realist reviews, appraisal with relevance, rigor and richness is the noted procedure to appraise papers included in this realist review.
  A sentence has been added to this section to state the procedure if a disagreement between MJN and ÉNS occurs.
  The appraisal of included paper is discussed in stage four of the process, but as already stipulated above, the Crowe Critical appraisal Tool was used due to its continuous utilisation in other realist reviews published in the literature.
  Given the qualitative nature of this review, reporting inter-rater reliability is not necessary.
- **Step IV doesn’t specify whether pilot testing of the extraction form will be done. Reliance on a single reviewer (MJN) for extraction, with supervisory oversight, carries a risk of bias.**
- **Response: **Thank you for this comment. A sentence or two has been added to stage four to reflect this.
  As noted above, this review will not be a systematic review and as such, yes, bias is more likely to be present, but in qualitative research bias is countered by strong reflexivity. However, the review is more concerned with the construction of mechanisms rather than bias as realist reviews are theory heavy. 
- **Step V, the process described could produce descriptive categories rather than true realist synthesis. Explicitly describe the use of retroductive reasoning, provide examples of how initial CMOs will evolve into middle-range theories, and consider external validation beyond the author panel.**
- **Response: **Thank you for this comment. Although we acknowledge your comment, we disagree with the statement that the process as outlined in stage 5 could produce descriptive categories. The process outlined in stage 5 is to support the development of refined initial programme theories and not the creation of middle-range programme theories which actually would require a realist evaluation in order for this to occur.
  External validation will occur through the expert panel as these are national experts on the implementation of CHIME into Irish services – where the overarching study is based. The review does not have funding to include international experts in the field.
  Stage 5 already discusses the retroductive reasoning to create refined IPTs. However, a sentence has been added to make this more visual.
- **Step VI, add transparency on how independence of critique will be preserved, and describe dissemination beyond academic journals (e.g., co-produced outputs for service users).**
- **Response: **The dissemination beyond academic journals is already described at the end of stage 6: *“* *In addition, the results of this proposed realist review may also be presented through other means, including conferences and recovery workshops delivered by recovery college personnel. This will be made possible through the Recovery and Engagement Programme Manager in charge of the recovery education workstream of MHER.”*
  Reporting bias will be contained through a process of reflexivity and journaling as the realist review process unfolds. This will be further noted in stage 6 of the process.
- **Figure 2 uses “Inductive Reflexive Thematic Analysis” under synthesis. This terminology is more common in qualitative research, but realist reviews emphasize retroductive reasoning and CMO refinement.**
- **Response: **Thank you for this comment. The wording of Figure 2 has been reworded to reflect your suggestion.
- **The current wording may mislead readers about analytic methods. This figure presents the steps as linear, but realist reviews are inherently iterative (moving back and forth between theory building, searching, and refinement). This could misrepresent the cyclical nature of realist reviews. Some steps (e.g., “Upload onto NVivo for Analysis”) are tool-specific, which may not apply universally and make the figure feel like a workflow chart rather than a generalizable method. Meanwhile, other important elements (e.g., stakeholder engagement, retroductive reasoning, theory testing vs. refinement) are missing. Advisory panel engagement should appear not just at the start, but also during synthesis/validation.**
- **Response: **These amendments to Figure 2 have now been made. Thank you for reminding us to include these elements in the Figure and for your guidance regarding the cyclical nature of realist reviews which should now be demonstrated in Figure 2.
- **Table 1 provides a useful but overly simplistic starting point for realist program theory development. It will benefit if you clarify distinctiveness by ensuring each CHIME domain’s If…Then statement highlights unique mechanisms. Add one illustrative case or a brief exemplary per domain to ground the theory.**
- **Response: **Thanks for this comment. Although we respect your perspective regarding table 1, we find it important to include as is as this demonstrates to you, the reader/reviewer where the review started from. The If… then… statements as they stand do show each element of the CHIME framework but at this point the if… then… statements may not have a clear mechanism attached. This comes with further refinement of the if… then… statements into initial IPTs.
  We also think an illustrative case or brief exemplary per domain is not required as these domains are already well documented in the existing literature.
- **Table 2 shows the evolution into draft CMOs. I recommend improvement for it.  The context should add diversity in settings (community vs. inpatient), roles (peer vs. clinician support), and cultural/systemic factors. Mechanisms should frame them as resources + reasoning (what is offered + how it is interpreted), e.g., “Access to peer groups (resource) triggers recognition of shared experience (reasoning)”. Outcomes should be more diverse by going beyond re-stating mechanisms, e.g., linking hope, such as increased engagement, reduced suicidality, and improved functioning.**
- **Response: **Thank you for this comment. This suggestion has been utilised in the actual realist review itself. However, although acknowledging the usefulness of the stated way to create IPTs, at this stage, due to advancement of the review process itself, making these amendments is now deemed impossible. However, the authors would like to thank you for raising this comment and wish to reassure you that this will be added to the review itself and be noted in the supplementary material as a deviation from the present protocol.
- **Table 3 remains too generic, tautological, and individual-focused. With richer contextualization, sharper mechanism articulation, and more concrete outcomes, it could provide a robust foundation for theory testing in the review.**
- **Response: **Thank you for this comment. The IPTs within table 3 cannot be changed as these IPTs have been approved by the expert panel to test within the actual realist review. However, what you have commented on is actually taking place within the review itself.
- **Table 4-6, Search strings like “Mental Health” OR “Mental Disorders” yield millions of hits (e.g., 6.5M in PubMed). This risks irrelevance and screening overload. It might indicate poor precision in early steps. Lack of Grey Literature Strategy in These Tables. Excluding pre-2011 studies may miss early CHIME conceptual development or precursors. Needs a stronger justification than “CHIME was published in 2011.**
- **Response: **Thank you for this comment. Table 4-6 were developed with the expertise of a librarian. Although we acknowledge your concern, we have already reached a stage in the review where we are extracting IPTs from included paper. As such, this comment can no longer be actioned upon.
  As noted above, a sentence or two has been added to state the process of grey literature searching as described by Godin and colleagues.
  A justification for excluding pre-2011 data has already been provided in the comments above.
- **Table 7, please reconsider including quantitative studies that explore mechanisms (e.g., mediation, regression, factor analysis). Provide a rationale for excluding dual diagnosis/addiction populations or consider including them with sensitivity analysis.**
- **Response: **Thank you for this comment. A justification has already been given as to why quantitative studies were excluded from this review.
  A rationale for excluding dual diagnosis/addiction populations has already been noted in the review. Here it states that they were excluded *“…* *because of the difficulties in distinguishing changes relating and not relating to mental health and illness alone*.”
